# Prognosis and risk factors for *ASXL1* mutations in patients with newly diagnosed acute myeloid leukemia and myelodysplastic syndrome

**DOI:** 10.1002/cam4.6871

**Published:** 2023-12-26

**Authors:** Liqing Yang, Xiaoyu Wei, Yuping Gong

**Affiliations:** ^1^ Department of Hematology, West China Hospital Sichuan University Chengdu Sichuan China; ^2^ Department of Hematology Fujian Medical University Union Hospital, Fujian Medical University Fuzhou Fujian China

**Keywords:** acute myeloid leukemia, *additional sex combs like 1* (*ASXL1*), myelodysplastic syndrome, prognosis, risk factor

## Abstract

**Objective:**

The objective of the study was to determine the prognosis and risk factors for *additional sex combs like 1* (*ASXL1*) mutations in patients with acute myeloid leukemia (AML) and myelodysplastic syndrome (MDS).

**Population and Methods:**

This retrospective study enrolled 219 adult patients with newly diagnosed AML and MDS, who were treated in West China Hospital from October 2018 to January 2022. The primary clinical outcome was evaluated by overall survival (OS) followed up to January 2023. Kaplan–Meier analysis and Cox multivariate regression analysis were performed to identify potential prognostic parameters in patients with *ASXL1* mutations (mt).

**Results:**

A total of 34 (15.53%) *ASXL1* ^*mt*^ were detected, which occurred more frequently in the elderly and MDS cohorts (*p* < 0.001). Significantly lower blasts% (*p* < 0.001) and higher frequencies of mutant *RUNX1*, *SRSF2*, *STAG2*, *EZH2*, and *SETBP1* (*p* < 0.02) were observed in the *ASXL1* ^mt^ cohort. Patients with *ASXL1* ^*mt*^ manifested with a worse complete remission rate (*p* = 0.011), and an inferior OS was shown in subgroups with MDS, co‐mutations of *RUNX1*, *SRSF2*, or *NRAS*, as well as mutations in G646W (*p* < 0.05). Multivariate analysis considering age, diagnosis, co‐mutations, and mutation site confirmed an independently adverse prognosis of mutations in G646W (HR = 4.302, 95% CI: 1.150–16.097) or *RUNX1* co‐mutations (HR = 4.620, 95% CI: 1.385–15.414) in the *ASXL1* ^*mt*^ cohort.

**Conclusion:**

Our study indicated that mutations in G646W or *RUNX1* co‐mutations are closely associated with a dismal clinical outcome in patients with AML and MDS harboring *ASXL1* ^mt^. Considering the poor prognosis and risk factors in patients with *ASXL1* ^mt^, more available treatments should be pursued.

## Graphical abstract

A retrospective study was conducted to shed light on potential prognostic risk factors in patients with AML and MDS carrying *ASXL1* mutations. Significantly, mutations in G646W and *RUNX1* co‐mutations were independently associated with inferior survival.

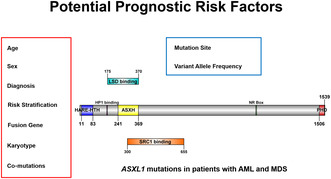

## 1 INTRODUCTION

Acute myeloid leukemia (AML) is the most common myeloid neoplasm worldwide with high heterogeneity, and myelodysplastic syndrome (MDS) is characterized by a high risk of transformation into AML. Although MDS is a different myeloid neoplasm from AML, there are some similarities in genetic mutant profiles and treatments,^1^ especially the increased blasts (IB) subtype. Thanks to advanced technologies, cytogenetic and molecular genetic features play a unique role in risk stratification and individualized treatment of myeloid neoplasms.^2^ As an enhancer of trithorax and polycomb (ETP), *additional sex combs like* (*ASXL)* family displays a biphasic effect on activating or silencing *homeobox A cluster* (*HOXA*) in Drosophila.^3^ The normal expression of *ASXL1* promotes the self‐renewal and differentiation of hematopoietic stem cells, which contributes to maintaining normal hematopoiesis.^3^ Associated with a dismal clinical outcome, *ASXL1* somatic mutations (mt) were reported in a variety of myeloid neoplasms^4^; while pathogenic germline mutations were more frequent in Bohring–Opitz syndrome, characterized by severe congenital deformity.^5^ Scattered cases concerning the presence of *ASXL1* germline mutations were also observed in familial hematological malignancies.^6^, ^7^ *ASXL2* was identified as a tumor suppressor, and deficiency in mice manifested severe impaired hematopoiesis in contrast to *ASX1* aberrations.^8^ A previous study revealed that *ASXL2* somatic mutations promoted leukemogenesis driven by *AML1::ETO* via regulating transcriptional effects.^9^ A higher frequency of *ASXL2* somatic mutations as well as a better prognosis was observed in patients with AML with the presence of *AML1::ETO*.^10^ Unlike *ASXL 1/2*, *AXSL 3* expression is restricted to certain tissues and somatic mutations are rare events in myeloid neoplasms.^3^, ^11^

**FIGURE 1:**
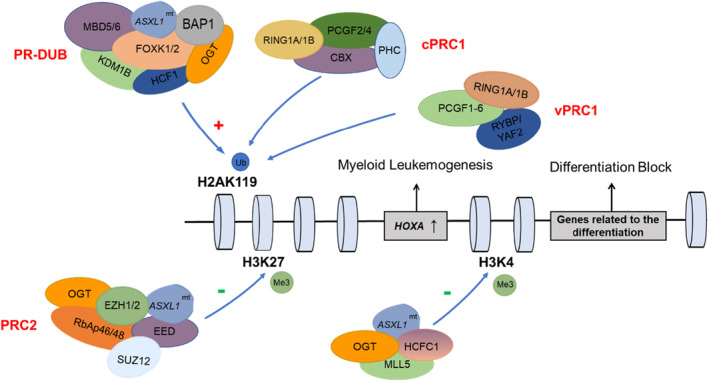
Pathogenic mechanism of ASXL1 aberration.

**FIGURE 2:**
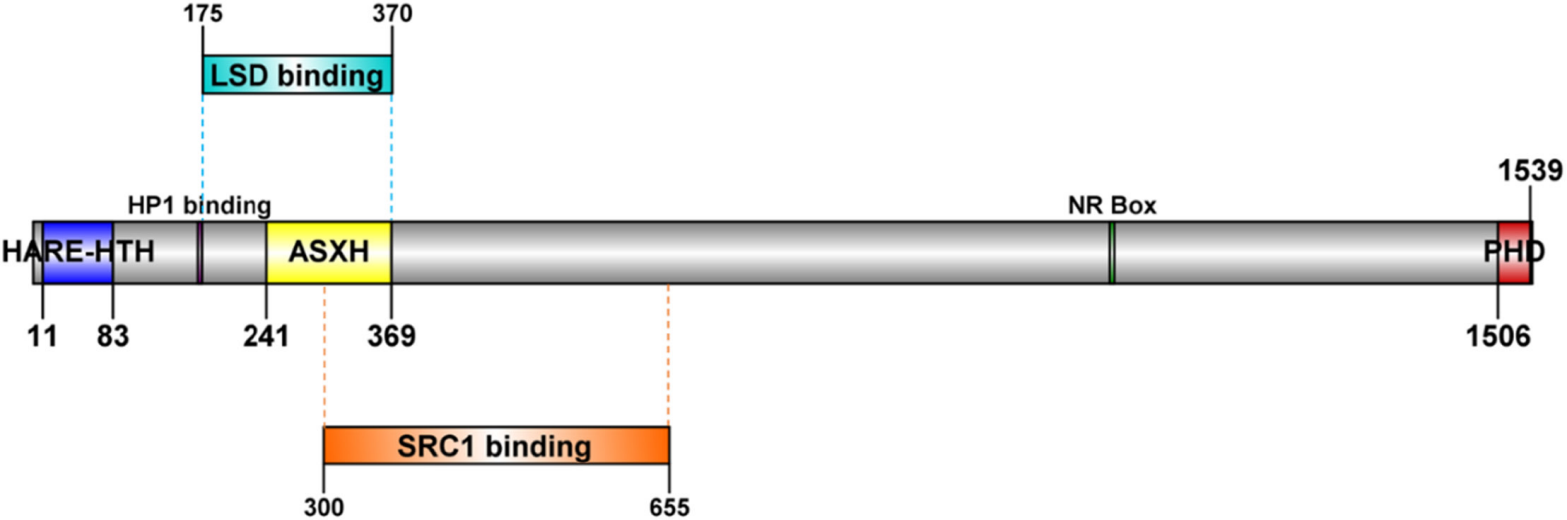
Schematic diagram of wild‐type ASXL1 (1541 aa).

The involvement of aberrant histone modifications and dysregulated transcription in the pathogenesis of *ASXL1* ^mt^ is widely recognized (Figure 1).^3^ ASXL1 variants enhance the catalysis of breast cancer 1 associated protein 1 (BAP1), leading to abnormal deubiquitination of histone H2AK119 mediated by polycomb repressive complex 2 (PRC2).^12^ Additionally, the overexpression of HOXA may be attributed to the hypomethylation of histone H3K27, which occurs due to the reduced enrichment of main components of PRC2 caused by ASXL1 depletion.^13^ Moreover, disruption of the interaction between wild‐type ASXL1 and a complex of O‐linked β‐N‐acetylglucosamine transferase and host cell factor C1 (OGT/HCFC1) results in the inhibition of histone H3K4 trimethylation, which in turn hampers the transcription of genes associated with hematopoietic stem cell differentiation.^14^ *ASXL1* is situated on chromosome 20q11.21 and encodes a protein consisting of 1541 amino acids, comprising three main domains (Figure 2).^3^, ^4^ *ASXL1* ^mt^ are frequently observed in exon 12, and manifest as heterozygous frameshift or nonsense mutations, leading to a C‐terminal truncated protein.

Among myeloid neoplasms, *ASXL1* ^mt^ were primarily detected in patients with chronic myelomonocytic leukemia (CMML) at a frequency of 40%–50%, and the occurrence rates in the MDS and AML cohorts were 11%–21% and 5%–11%, respectively.^3^ *ASXL1* ^mt^ are common in clonal hematopoiesis and considered to be relatively early events in leukemogenesis,^15^ with increased dominant clones in secondary AML (sAML) post MDS.^16^, ^17^ According to the 2022 European leukemia network (ELN) guidelines, *ASXL1* is categorized as AML with myelodysplasia‐related genes.^18^ Based on abundant real‐world data,^14^, ^19^, ^20^, ^21^, ^22^, ^23^ adverse prognosis of *ASXL1* ^mt^ in patients with AML was claimed early in the 2017 ELN guidelines.^24^ However, due to high heterogeneity in patients with myeloid neoplasms, further investigation is needed to determine potential prognostic factors in the *ASXL1* ^mt^ cohort to improve clinical treatment and management. Herein, with the aim of enriching prognostic markers of *ASXL1* ^mt^, we conducted a retrospective study to analyze the prognosis and risk factors for *ASXL1* ^mt^ in patients with AML and MDS, including age, sex, risk stratification, fusion gene, karyotype, co‐mutations of high frequencies, variant allele frequency (VAF), and mutation site of *ASXL1*.

## 2 POPULATION AND METHODS

### 2.1 Criteria for patient selection

During the period from October 2018 to January 2022, a total of 483 adult patients were treated in West China Hospital and participated in the detection of gene mutations in myeloid neoplasms conducted by West China Kang Shengda Laboratory Company. Except for patients without necessary clinical data (*n* = 82), exclusion criteria included the following: (1) diagnosis excepted AML and MDS based on the 5th World Health Organization (WHO) diagnostic criteria^15^ (*n* = 85); (2) non‐newly diagnosed cases (*n* = 23); 3) treatment‐related AML arising post solid tumors (*n* = 10); (4) acute promyelocytic leukemia (APL) in the AML cohort (*n* = 9); (5) other subtypes instead of IB in the MDS cohort (*n* = 35); and (6) *ASXL1* germline mutations or somatic mutations with undetermined significance (*n* = 20). Finally, 219 patients who were newly diagnosed with AML (not APL) or MDS‐IB were enrolled in this retrospective study. Clinical data were recorded including basic information, routine examination, molecular and genetic detection, treatment, and survival status, with a follow‐up up to January 2023. This study was approved by the ethics committee of Sichuan University and performed in accordance with the 1964 declaration of Helsinki. A written informed consent for data collection and publication was obtained from each patient or authorized agent prior to the enrollment of the study.

### 2.2 Genetic analysis

Approximately 3 mL of peripheral blood (PB) or bone marrow (BM) with ethylenediaminetetraacetic acid (EDTA) anticoagulation was collected from each participant to extract genomic DNA with the Automated Nucleic Acid Purification Apparatus HF16 Plus (GONCERT). Authorized to West China Kang Shengda Laboratory Company, a panel of 34 high‐frequency gene mutations (Table S1) was assessed by next‐generation sequencing (NGS) with NextSeq 550 sequenator (Illumina). The detection covers point, insertion, and deletion mutations in selected exons as well as nearby introns within 10 base pairs. The transcript of *ASXL1* is NM_015338, with detection focused on exon 12. Bioinformatics analysis of raw sequencing data was performed in the NCBI, COSMIC, dbSNP, and ClinVar databases to determine the pathogenic mutation site. Nomenclature of sequence variants refers to the 2015 American College of Medical Genetics and Genomics (ACMG) guidelines.^25^ In addition, with mononuclear cells collected from another EDTA anticoagulant tube, RNA was extracted using the TRIzol technique by RNAiso Plus Kit (TAKARA). Fusion genes were identified by real‐time quantitative PCR with Leukemia Fusion Gene Diagnostic Kits (MyGenostics), with screening a minimal panel including *AML1::ETO*, *CBFβ::MYH11*, *BCR::ABL1*, *PML::RARα*, *MLL::AF4/AF6/AF9*, *E2A::PBX1*, *SIL::TAL1*, and *TEL::AML1*.

### 2.3 Statistical analysis

Statistical analysis and graphical illustration were performed with SPSS (version 25.0), IBS (version 1.0) and Xiantao Academic analysis (https://www.xiantaozi.com/). Mann–Whitney test was used for the comparisons of measurement data between groups, in contrast to chi‐square test or Fisher's exact test for count data. Potential parameters were considered to determine the prognostic significance in patients with *ASXL1* ^mt^, including age, sex, diagnosis, risk stratification, white blood cells (WBC) count, VAF, mutation site, karyotype, fusion genes, and co‐mutations of high frequencies. The optimal cutoff value of continuous variables for clinical outcomes was determined with receiver operating characteristic (ROC) curve, or a median value if unavailable. Overall survival (OS) was defined as the period from the start of diagnosis to death or time of last follow‐up. The difference in OS was estimated by the Kaplan–Meier method with the log–rank test, and multivariate analysis was conducted in a forward LR Cox proportional hazards regression model. A two–sided *p* < 0.05 was considered a significant difference.

## 3 RESULTS

### 3.1 Clinical characteristics

*ASXL1* ^mt^ were detected in a total of 34 (15.53%) patients, and the clinical characteristics are presented in Table 1. Compared with the wild–type (wt) control, patients with *ASXL1* ^mt^ exhibited an older onset age (*p* < 0.001) with an average age of 60.41 ± 14.03 years, and a larger proportion of the MDS cohort (*p* < 0.001). Further details of the morphological subtype and risk stratification are provided in Table S2. Additionally, patients harboring *ASXL1* ^mt^ manifested significantly lower percentages of blasts in both PB and BM before treatment (*p* < 0.001), along with relatively elevated levels of uric acid (UA) (*p* = 0.046), and smaller eGFR values (*p* < 0.001). In addition, blasts% only exhibited a statistically significant difference in the AML cohort (*p* ≤ 0.020), rather than in the MDS cohort. Similar to the control group, a majority of patients with *ASXL1* ^mt^ presented with a normal karyotype (52.38%) and no specific fusion genes (77.27%). In the *ASXL1* ^mt^ cohort, complex karyotypes were observed in two individuals and *AML1::ETO* was the only recorded positive fusion gene. Independently, 13 (38.24%) and 12 (35.29%) patients with *ASXL1* ^mt^ lacked a report of karyotype or fusion genes due to economic reasons or unexplained loss. Meanwhile, the situations above in the control cohort were 32 (17.30%) and 39 (21.08%), respectively.

**TABLE 1:** Clinical characteristics based on *ASXL1* mutation status.

	*ASXL1* ^wt^ (*n* = 185)	*ASXL1* ^mt^ (*n* = 34)	*p*‐value
Essential features			
Age (years)	48.00 (18–83)	60.41 ± 14.03	<0.001^§^
Male	89 (48.11)	15 (44.12)	0.712^§^
Patients with AML	157 (84.86)	19 (55.88)	<0.001^§^
Routine examinations			
HGB (×10^12^/L)	75.50 (29–141)	73.79 ± 24.40	0.153^‡^
WBC (×10^9^/L)	8.95 (0.31–368.54)	3.64 (0.97–136.29)	0.087^‡^
PLT (×10^9^/L)	40.00 (1–1048)	45.00 (5–614)	0.259^‡^
Blast in PB (%)	25.00 (0–97)	0 (0–89)	<0.001^‡^
Blast in BM (%)	51.00 (5.80–97.00)	26.95 (6.00–80.00)	<0.001^‡^
ALT (IU/L)	17.00 (5–175)	14.00 (6–107)	0.736^‡^
ALB (IU/L)	40.90 (26.20–52.00)	40.65 (22.00–46.70)	0.714^‡^
LDH (mmol/L)	309.50 (118–9013)	284.50 (134–3466)	0.251^‡^
UA (umol/L)	285.00 (60–917)	373.74 ± 159.05	0.046^‡^
eGFR (mL/min/1.73 m^2^)	101.70 (37.34–153.77)	81.08 ± 26.71	<0.001^‡^
Cytogenetic characteristics			
Normal karyotype	82 (53.59)	11 (52.38)	1.000^§^
Complex karyotype	22 (14.38)	2 (9.52)	0.732^§^
No fusion gene	103 (70.55)	17 (77.27)	0.619^§^
*AML1::ETO* (+)^a^	22 (15.07)	5 (22.73)	0.534^§^
Co‐mutations			
*RUNX1*	19 (10.27)	10 (29.41)	0.005^§^
*TET2*	23 (12.43)	10 (29.41)	0.018^§^
*STAG2*	8 (4.32)	10 (29.41)	<0.001^§^
*SRSF2*	4 (2.16)	8 (23.53)	<0.001^§^
*NRAS*	21 (11.35)	6 (17.65)	0.391^§^
*EZH2*	7 (3.78)	6 (17.65)	0.007^§^
*DNMT3A*	36 (19.46)	5 (14.71)	0.636^§^
*IDH2*	23 (12.43)	3 (8.82)	0.757^§^
*U2AF1*	6 (3.24)	3 (8.82)	0.300^§^
*SETBP1*	1 (0.54)	3 (3.82)	0.009^§^
*BCOR*	15 (8.11)	3 (3.82)	0.923^§^
*KIT*	12 (6.49)	2 (5.88)	1.000^§^
*TP53*	17 (9.19)	2 (5.88)	0.759^§^
*ZRSR2*	2 (1.08)	2 (5.88)	0.221^§^
*IDH1*	12 (6.49)	2 (5.88)	1.000^§^
*MPL*	0	1 (2.94)	0.155^¶^
*PTPN11*	13 (7.03)	1 (2.94)	0.607^§^
*PHF6*	3 (1.62)	1 (2.94)	1.000^§^
*EVT6*	1 (0.54)	1 (2.94)	0.710^§^
*CSF3R*	9 (4.86)	1 (2.94)	0.963^§^
*KRAS*	13 (7.03)	1 (2.94)	0.607^§^
*FLT3*	37 (20.00)	1 (2.94)	0.030^§^
*NPM1*	26 (14.05)	1 (2.94)	0.127^§^
*CEBPA*	30 (16.22)	0	0.006^¶^
Treatments and clinical outcomes^b^			

HMA‐included Therapy	62 (37.58)	13 (61.90)	0.037^§^
Allo‐HSCT	38 (20.54)	1 (2.94)	0.098^§^
Complete remission	124 (75.15)	10 (47.62)	0.011^§^
Relapse	53 (42.74)	3 (30.00)	0.651^§^
Death	77 (41.62)	14 (41.18)	1.000^§^

*Note*: The distribution of measurement data is represented by the mean ± standard deviation (SD) or median (range) if not conforming to normality, while count data are presented as number (%).

Statistical analysis: ^‡^Mann–Whitney test, ^§^(approximate) chi‐square test, ^¶^Fisher's exact test.

Abbreviations: ALB, albumin; Allo‐HSCT, allogeneic stem cell transplant; ALT, alanine transaminase; BM, bone marrow; eGFR, estimated glomerular filtration rate; HGB, hemoglobin; HMA, hypomethylating agent; LDH, lactate dehydrogenase; PB, peripheral blood; PLT, thrombocyte; UA, uric acid; WBC, white blood cell.

^a^ *AML1::ETO*, also known as *RUNX1::RUNX1T1*, which is originated from t(8;21)(q22;q22) chromosomal translocation and different from *RUNX1* point mutation, has been identified as a favorable genetic feature in patients with AML in 2022 ELN guidance.

^b^ The treatment referred to the first induced therapy, and subsequent treatments were not included.

### 3.2 Genetic characteristics

The median VAF of *ASXL1* ^mt^ was 42.15% (range 4.50%–62.92%). Among patients with *ASXL1* ^mt^, there were 29 (85.29%) frameshift mutations, four (11.76%) nonsense mutations, and only one insertion mutation (Table 2). A total of 12 sequence variants were detected, and G646WfsX12 was the most common amino acid change with a frequency of 47.06% (*n* = 16), followed by G635RfsX15 with a frequency of 20.59% (*n* = 7). Approximately 94.12% of patients carried concomitant mutations, while *RUNX1*, *TET2*, and *STAG2* were identified as the most frequent co‐mutations in 10 (29.41%) individuals, followed by *NRAS* (17.65%), *EZH2* (17.65%) and *DNMT3A* (14.71%). In addition, wild‐type *FLT3*, *NPM1*, and *CEBPA* seemed to be closely associated with *ASXL1* ^mt^. The frequencies of *RUNX1* (*p* = 0.005), *TET2* (*p* = 0.018), *STAG2* (*p <* 0.001), *SRSF2* (*p* < 0.001), *EZH2* (*p* = 0.007), *SETBP1* (*p* = 0.009), *FLT3* (*p* = 0.030), and *CEBPA* (*p* = 0.006) co‐mutations were significantly different in patients with *ASXL1* ^mt^ in contrast to those with *ASXL1* ^wt^. Further details of the mutant profiles in the *ASXL1* ^mt^ cohort are shown in Figure S1.

**TABLE 2:** Genetic characteristics in patients with *ASXL1* ^mt^ (*n* = 34).

Pt	Sex	Onset age (years)	Diagnosis	Risk stratification^a^	cDNA change	Amino acid change	VAF (%)	Karyotype	Fusion genes	Concomitant mutations
1	F	74	AML‐M2	Adverse	c.2465del	p.T822NfsX2	49.52	NA	Negative	*NRAS, TET2, SRSF2, RUNX1, STAG2*
2	F	71	MDS‐IB2	High	c.1934dup	p.G646WfsX12	43.10	47, XY, +8(20)	Negative	*EZH2, KRAS, NRAS, RUNX1, TET2, STAG2*
3	M	61	MDS‐IB1	High	c.1934dup	p.G646WfsX12	40.40	Normal	NA	*RUNX1, STAG2, ZRSR2, TET2*
4	M	82	MDS‐IB1	Intermediate	c.1934dup	p.G646WfsX12	43.00	Normal	NA	*BCOR, RUNX1, TET2, SRSF2*
5	M	83	MDS‐IB1	High	c.1774C>T	p.Q592X	45.30	NA	NA	*RUNX1, SRSF2, STAG2*
6	F	78	AML‐M4	Adverse	c.1934dup	p.G646WfsX12	38.40	NA	Negative	*BCOR, RUNX1, SRSF2*
7	M	65	MDS‐IB2	High	c.1772dup	p.Y591ins	42.40	Normal	Negative	*KIT, RUNX1, EZH2, PTPN11*
8	M	34	MDS‐IB2	Very High	c.1934dup	p.G646WfsX12	37.30	NA	Negative	*CSF3R, U2AF1, RUNX1*
9	M	49	MDS‐IB1	Intermediate	c.1934dup	p.G646WfsX12	36.10	NA	Negative	*RUNX1, PHF6, SF3B1, KMT2D*
10	F	74	AML‐M2	Adverse	c.1934dup	p.G646WfsX12	38.70	NA	NA	*CBL, DNMT3A, SETBP1, SRSF2*
11	F	45	MDS‐IB2	Very High	c.2464dup	p.T822NfsX11	50.00	NA	NA	*RUNX1, SETBP1, DNMT3A, ROBO2, RUNX1*
12	M	64	AML‐M2	Adverse	c.1900_1922del	p.G635RfsX15	50.90	Normal	Negative	*SRSF2, STAG2, TET2*
13	M	75	AML‐M2	Adverse	c.1934dup	p.G646WfsX12	39.20	Abnormal^b^	NA	*NRAS, TET2, ZRSR2, STAG2*
14	M	68	MDS‐IB1	Intermediate	c.3120del	p.A1041PfsX6	49.60	Normal	Negative	*STAG2, TET2, EZH2*
15	M	70	AML‐M5	Adverse	c.2057dup	p.C687VfsX31	45.50	Normal	Negative	*CBL, SRSF2, TET2*
16	M	58	MDS‐IB2	High	c.2269C>T	p.Q757X	47.40	NA	NA	*EVT6, EZH2, TET2, U2AF1, BCOR*
17	F	72	AML‐M2	Adverse	c.2338C>T	p.Q780X	8.00	NA	NA	*EZH2*
18	F	68	AML‐M5	Adverse	c.2866dup	p.L956PfsX14	28.60	Normal	Negative	*CBL, NPM1, TET2*
19	M	57	AML‐M2	Adverse	c.1934dup	p.G646WfsX12	39.10	Normal	Negative	*NRAS, IDH2, SRSF2, STAG2*
20	F	69	MDS‐IB2	Very High	c.1934dup	p.G646WfsX12	50.00	NA	Negative	*STAG2, BCOR*
21	F	57	MDS‐IB2	High	c.1900_1922del	p.G635RfsX15	48.00	Normal	Negative	*IDH2, STAG2*
22	M	66	AML‐M4	Adverse	c.1934dup	p.G646WfsX12	44.20	42~44, XY, ‐5, add(7) (q22), ‐9, ‐16, ‐17, +1~2mar[16/]46, XY[4]	Negative	*MPL, NRAS, PTPN11, TP53, EZH2*
23	M	59	AML‐M2	Adverse	c.1900_1922del	p.G635RfsX15	34.10	46, XY, t(8;21) (q22;q22) [1]/47, idem,+der(21)(8;21)[17]/46, XY[2]	*AML1::ETO*	*NRAS, ROBO1*
24	F	39	AML‐M4	Adverse	c.1900_1922del	p.G635RfsX15	43.30	Normal	Negative	*DNMT3A, IDH1*
25	F	66	AML‐M2	Adverse	c.1934dup	p.G646WfsX12	50.00	47, XX, +8[20]	Negative	*DNMT3A, IDH2*
26	F	50	AML‐M2	Adverse	c.1934dup	p.G646WfsX12	37.60	NA	NA	*DNMT3A, IDH1*
27	F	62	MDS‐IB1	High	c.1900_1922del23	p.G635RfsX15	9.10	NA	NA	*CBL*
28	F	36	MDS‐IB1	High	c.1934dup	p.G646WfsX12	17.50	Normal	NA	*U2AF1, ETV6*
29	F	65	MDS‐IB1	High	c.1774C>T	p.Q592X	35.40	NA	NA	*SETBP1*
30	F	70	AML‐M2	Adverse	c.1934dup	p.G646WfsX12	41.90	46, XX, del(7) (q32), t(8;21) (q22;q22) [20]	*AML1::ETO*	*KIT*
31	F	41	AML‐M2	Adverse	c.1900_1922del23	p.G635RfsX15	50.00	46,XX,t(8;21)(q22;q22)[19]/46,XX	*AML1::ETO*	*PHF6*
32	F	45	AML‐M4	Adverse	c.2279_2280dupAG	p.A761RfsX12	62.92	42‐45,XX,‐1, der(2) t(1;2) (q12;q36)	Negative	*TP53*
33	M	26	AML‐M4	Adverse	c.1900_1922del	p.G635RfsX15	4.50	45, X, ‐Y, t(8;21)(q22;q22) [18] /46, XY[2]	*AML1::ETO*	
34	F	55	AML‐M2	Adverse	c.1934dup	p.G646WfsX12	5.26	45, X, ‐X, t(8;21)(q22; q22)[20]	*AML1::ETO*	

Abbreviations: F, Female; M, Male; NA, not available. (amino acid) Y, tyrosine; G, glycine; W, tryptophan; R, arginine; T, threonine; N, asparagine; A, alanine; Q, glutarnine; P, proline; C, cystine; V, valine.

^a^ Risk stratification in the MDS cohort is based on the IPSS‐R criterion, and *ASXL1* ^mt^ are associated with an adverse prognosis in the AML cohort according to 2022 ELN guidelines.

^b^ Abnormal karyotype of pt13 was recorded in the electronic medical record system without available details.

### 3.3 Clinical outcomes

There were 20 (10.81%) and 13 (38.24%) patients who declined treatment in the control and *ASXL1* ^mt^ cohorts, respectively. The proportion of patients with *ASXL1* ^mt^ who received the hypomethylating agent (HMA)‐included regimen as initial therapy was 61.90%, which was statistically higher than that of patients with *ASXL1* ^wt^ (*p* = 0.037). In the *ASXL1* ^mt^ cohort, five patients received HMA alone, and two patients used combined treatment of HMA and homoharringtonine and cytarabine (HA), and combined treatment of HMA and idarubicin and cytarabine (IA) or daunorubicin and cytarabine (DA) or venetoclax (VEN) each presented in three patients. Traditional treatment without HMA including DA, IA, and HA was adopted in another six individuals. Further details of treatment in the control group are provided in Table S2. Only one patient was recorded with allo‐HSCT in the *ASXL1* ^mt^ cohort, in contrast to 38 (20.54%) individuals in the control group. After at least two rounds of induction chemotherapy, an apparently suboptimal complete remission (CR) rate was observed in patients with *ASXL1* ^mt^ compared to those with *ASXL1* ^wt^ (*p* = 0.011), while the rates of relapse or death barely presented differences. Moreover, only three individuals (two in the control group) complicated with central nervous system leukemia (CNSL) were observed.

**FIGURE 3:**
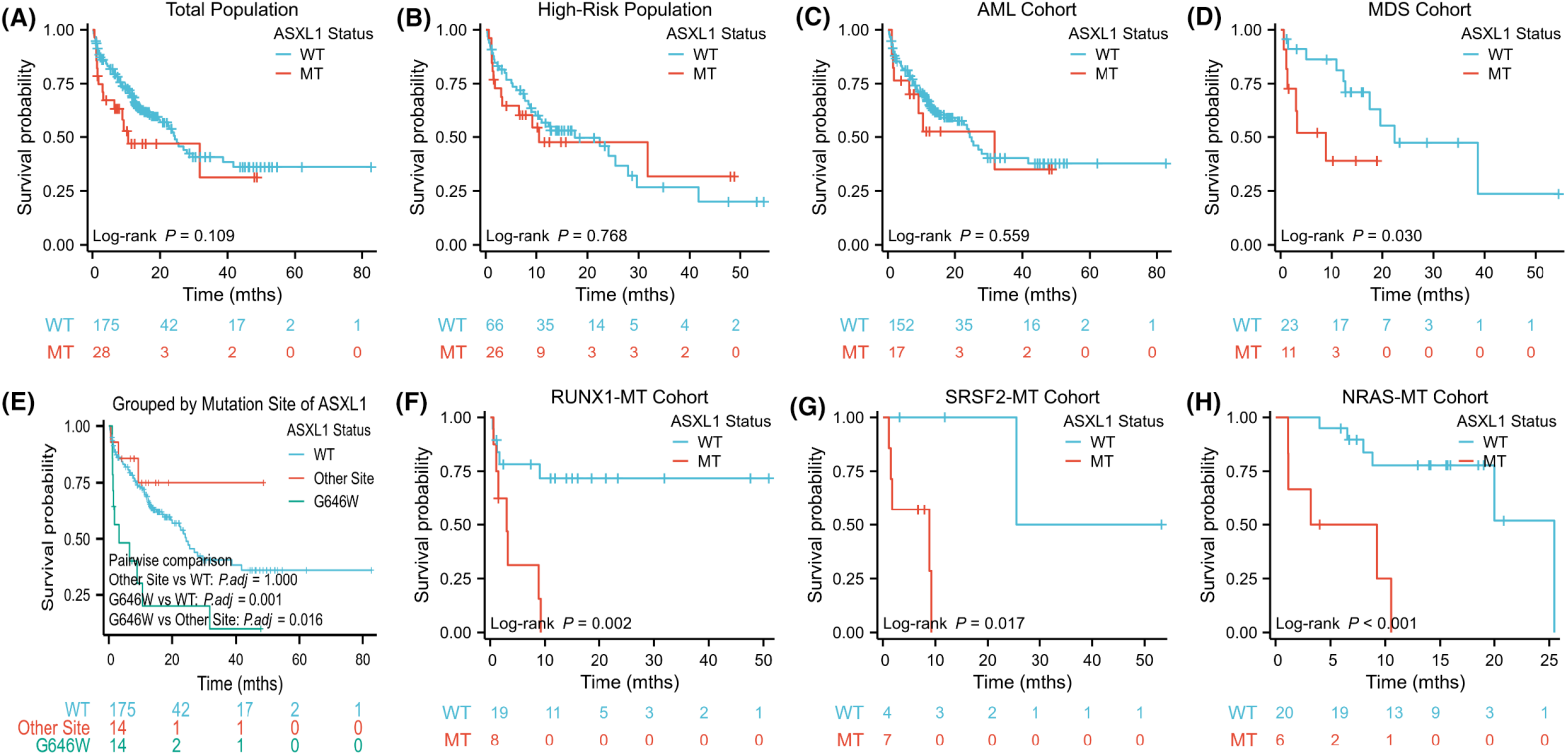
Survival analysis in total population and subgroups based on diagnosis, risk stratification, mutation site of *ASXL1* and co‐mutations of high frequencies.

The median OS for patients with *ASXL1* ^wt^ or *ASXL1* ^mt^ was 24.39 (range 20.45–28.33) months and 10.53 (range 0–28.45) months, respectively, without a significant difference (*p* = 0.109), nor in the high‐risk cohort who were considered to have adverse prognosis according to clinical guidelines^18^, ^26^ (*p* = 0.768). (Figure 3A,B). Despite no statistically significant difference in the AML cohort (*p* = 0.559), an inferior OS was observed in the MDS cohort carrying *ASXL1* ^mt^ (*p* = 0.030) (Figure 3C,D). Further univariate analysis of potential prognostic parameters was performed including age (58 years old as a cut‐off), sex, a large WBC count (≥35 × 10^7^/L), karyotype, fusion gene (the presence of *AML1::ETO*), VAF (the median value as a cutoff), mutation site of *ASXL1*, treatment (whether HMA includes) (Figure S2), and co‐mutations of high frequencies (Figure S3). Kaplan–Meier analysis indicated worse survival in patients with mutations in G646W (*p* = 0.001) and co‐mutations of *RUNX1* (*p* = 0.002), *SRSF2* (*p* = 0.017), or *NRAS* (*p* < 0.001) in the *ASXL1* ^mt^ cohort (Figure 3E–H). Considering the potential prognostic markers including age, diagnosis, risk stratification, karyotype, fusion gene, and co‐mutations of *RUNX1*, *SRSF2*, or *NRAS*, multivariate Cox regression analysis was conducted and showed that older age remained the only significant variable in the total population (hazard ratio [HR] = 2.784, 95% confidence interval [CI]: 1.613–4.806). To further explore aggravated factors of prognosis in patients with *ASXL1* ^mt^, multivariate Cox regression analysis was performed and demonstrated independently adverse markers of mutations in G646W (HR = 4.302, 95% CI: 1.150–16.097) or *RUNX1* co‐mutations (HR = 4.620, 95% CI: 1.385–15.414) when variables including age, sex, diagnosis, mutation site, and co‐mutations were considered (Figure 3A). Risk stratification was not included in the model due to a large proportion of the high‐risk group in patients with *ASXL1* ^mt^, which is categorized into adverse genetic features in the AML cohort (2022 ELN). *RUNX1* co‐mutations with *ASXL1‐*G646W were observed in five individuals, and a closely inferior OS remained compared with the control group (*p* = 0.056) (Figure S4).

## 4 DISCUSSION

In this study, *ASXL1* ^mt^ were detected in 15.53% of patients with AML and MDS, in accordance with previous research. We found that *ASXL1* ^mt^ was relatively common in the elderly cohort and the MDS cohort, without a difference in the sex distribution, while a higher frequency in the male cohort was reported in previous studies.^20^, ^21^, ^23^, ^27^ Similarly, a significantly lower blasts% in PB or BM was exhibited in the *ASXL1* ^mt^ cohort,^14^ while mildly elevated levels of UA and smaller eGFR values were accidentally observed in our study. A smaller WBC count was also regarded as an apparent clinical feature of patients with *ASXL1* ^mt^,^14^, ^21^ which presented a tendency in our cohort without significance. No significant difference was shown in cytogenetic characteristics including karyotype and fusion gene in our study. Aberrant karyotypes were reported to be associated with *ASXL1* ^mt^,^20^, ^28^ especially intermediate‐risk karyotypes in the MDS cohort.^19^ Consistently, *AML1::ETO* was identified as a common fusion gene in patients with *ASXL1* ^mt^.^29^ *ASXL1* ^mt^ in myeloid neoplasms were considered to be ubiquitously accompanied by mutations of *RUNX1*, *SRSF2*, *STAG2*, *NRAS*, or *IDH2*, in addition to wild‐type *NPM1* and *FLT3*,^14^, ^21^, ^22^, ^28^ which was aligned with the mutation profile in our cohort. Moreover, our data also indicated statistically different distributions of *TET2*, *EZH2*, *SETBP1*, and *CEBPA* mutations between the two groups.

Early in 2010, *ASXL1* ^mt^ were reported to be associated with a dismal clinical outcome in adult patients with primary AML.^28^ Subsequent research indicated an independent adverse prognosis of *ASXL1* ^mt^ in the MDS cohort.^19^ In our study, no significantly differential survival was observed in the total population based on *ASXL1* mutation status, nor in the AML cohort, while an inferior OS was observed in the MDS cohort carrying *ASXL1* ^mt^. In patients with *ASXL1* ^mt^, the median age of the AML cohort and MDS cohort was 66 (26–78) years and 62 (34–83) years, respectively. While the median age of aforementioned cohorts in patients with *ASXL1* ^wt^ was 48 (15–81) years and 54 (14–83) years, correspondingly. An apparent discrepancy was supposed to be observed in the AML cohort when giving priority to the age distribution. The reasons for no significance in patients with AML might be a result of individual variation due to a small sample and significantly higher blasts% in the wild‐type group with a large proportion of FAB‐M1 subtype (Table S2). Previous studies indicated a high frequency of *ASXL1* ^mt^ in patients with MDS, which was associated with an increased risk of transformation into AML.^19^ Susceptibility to leukemic transformation was identified to be elevated by additional mutations including *SETBP1*, *RUNX1*, or *NRAS*.^30^, ^31^, ^32^ Moreover, *ASXL1* ^mt^ in the AML cohort was reported to be more frequent in patients with sAML^21^, ^23^ and AML with myelodysplasia‐related changes (MRC).^33^ The diagnosis of enrolled patients in our study refers to their primary diagnosis before induction treatment. We observed five (17.86%) and one (6.66%) patient with MDS transforming into AML in the control cohort and the *ASXL1* ^mt^ cohort, respectively. In addition, there were independently seven (4.46%) and one (5.26%) patients with sAML post MDS in the two groups. Certain cases of omission diagnosis, limited follow‐up period or a small sample should be responsible for a suboptimal transformation rate in our study. *ASXL1* ^mt^ in the de novo AML cohort was reported to be associated with a lower CR rate and a short survival,^27^ while an inferior OS was observed in the sAML cohort with *SRSF2* co‐mutations.^34^ Further studies on the prognosis analysis between de novo AML and sAML are warranted.

Although *ASXL1* ^mt^ have been widely considered as an adverse prognostic marker in myeloid neoplasms, risk factors contributing to a worse clinical outcome still merit further exploration. A meta‐analysis in the AML cohort indicated that cytogenetically normal (CN) or elderly patients with *ASXL1* ^mt^ manifested an inferior OS.^35^ Furthermore, *ASXL1* ^mt^ in elderly patients with CN‐AML were associated with significantly unfavorable CR rates and clinical outcomes.^14^ No significant survival difference based on age or karyotype was presented in our cohort due to a small sample size and a ratio of unavailable data. Yi F, et al. reported that a larger WBC count (≥ 50 × 10^9^/L), the absence of *AML1::ETO* and co‐mutations of *FLT3‐ITD* or *RUNX1* were other hazard signs of prognosis in patients with *ASXL*1^mt^.^29^ In our study, a smaller WBC count was observed in the *ASXL*1^mt^ cohort, and no significance of OS presented in patients with a large WBC count (≥ 35 × 10^9^/L), which might be a result of a small sample and setting of the cut‐off value. A tendency of relatively better survival was exhibited in our study in patients carrying *ASXL*1^mt^ with the presence of *AML1::ETO*, and more real‐world data are needed. Since *AML1::ETO* was the only positive result in our cohort, the effects of other fusion genes remain for further discussion. Prognosis analysis considering concomitant genes was conducted and showed a significantly shorter survival in co‐mutations of *RUNX1*, *SRSF2*, or *NRAS* in patients with *ASXL1* ^mt^. As mentioned earlier, *RUNX1* and *NRAS* mutations were driving factors of leukemic transformation, while *SRSF2* was identified as a hazard for increased mortality in patients with AML harboring *ASXL1* ^mt^.^34^ A shorter survival was also observed in patients suffering from myeloid neoplasms when *ASXL1* ^mt^ were complicated with mutations of *SF3B1*, *SETBP1*, or *JAK2‐V617F*.^36^, ^37^, ^38^ Similar to *ASXL1*, *RUNX1* has also been categorized into adverse prognostic genes in the AML cohort according to the 2022 ELN guidelines.^18^ A high frequency and a significantly inferior survival of *RUNX1* co‐mutations in patients with AML or MDS carrying *ASXL1* ^mt^ was observed in previous studies, both in the elderly cohort^39^, ^40^ and the younger cohort.^23^ Our results demonstrated that *RUNX1* co‐mutations in the *ASXL1* ^mt^ cohort were independently associated with an adverse prognosis, while inapparent significance of OS presented in co‐mutations of *SRSF2* or *NRA*S in multivariate analysis was possibly due to limited available data.

Survival analysis was further performed to clarify prognostic markers in genetic features. Increased VAFs of driving genes was identified as a predictor of leukemic transformation in patients with MDS or myeloproliferative neoplasms (MPNs),^41^, ^42^ and a higher VAF was reported to be involved in the poor survival of patients with newly diagnosed AML.^43^ Our data did not show a significant influence of VAF on the prognosis of patients with *ASXL*1^mt^, which might be a result of a different cutoff value, a combined study population of MDS and AML and a small sample size. Frameshift mutations were considered to be independently associated with a worse prognosis in patients with MDS harboring *ASXL1* ^mt^.^19^ Nevertheless, another study indicated an inferior OS of *ASXL1* frameshift mutations in the CMML cohort without significance in the MDS cohort.^44^ Since only two available data were excluded in the *ASXL1* frameshift mutation group, survival analysis based on mutation type was not conducted. Instead, our study provided a real‐world clinical data about dismal survival due to *ASXL1‐*G646W mutations in patients with AML and MDS for the first time. Located on the steroid receptor coactivator 1 (SRC1) binding region of the ASXL1 protein,^45^ G646W mutations are the most frequent amino acid change, and have been recently identified as a bona fide somatic mutation instead of an artifact.^46^, ^47^ Previous research indicated that *ASXL1‐*G646W mutations were associated with shorter OS and leukemia‐free survival (LFS) in patients with primary myelofibrosis (PMF).^48^ Furthermore, an animal model demonstrated that *ASXL1*‐G643W mutations (the most common mutation according to the authors) contributed to the development of *CEBPA*‐driven AML, which is involved in the resistance to chemotherapy.^23^, ^49^ Considering the adverse prognostic parameters identified in our study, a risk model including elderly age, and co‐mutations of *RUNX1* and *ASXL1‐*G646W could be reasonably presumed in patients with MDS, and large‐sample multicenter studies are needed.

Current evidence has showed that *ASXL1* ^mt^ conserved as an adverse prognostic marker in HMA‐included therapy.^50^, ^51^ In our cohort, a poor response to treatment was observed in patients with *ASXL1* ^mt^. A study indicated that increased hits of risk factors were associated with a deteriorated prognosis of patients with *ASXL1* ^mt^, and early allogeneic hematopoietic stem cell transplantation after the first CR was proposed.^29^ Presently, no specific targeted therapies concerning *ASXL1* ^mt^ have been recommended for patients with myeloid neoplasms according to updated clinical guidelines.^15^ Patients with newly diagnosed AML harboring *ASXL1* ^mt^ who are eligible for intensive chemotherapy are more likely to receive combined treatment of HMA and VEN approved by the Food and Drug Administration (FDA).^52^ Significantly, although VEN‐based therapy has been identified as an effective induced therapy in elderly patients with newly diagnosed AML, apparent resistance in monocytic AML should be concerned.^53^, ^54^ In addition, *BAP1* was considered a potential therapeutic target in patients with *ASXL1* gain‐of‐function mutations, with high sensitivity in vitro and an improved OS in a patient‐derived xenograft mouse model.^55^, ^56^ Considering *ASXL1* ^mt^ are more frequent in the elderly and patients with adverse cytogenetic and molecular features, more available treatments should be pursued.

## 5 CONCLUSION

To summarize, our results showed that patients with *ASXL1* ^mt^ manifested with a worse CR rate, and an inferior OS was observed in patients with MDS, co‐mutations of *RUNX1*, *SRSF2*, or *NRAS*, and mutations in G646W. Multivariate analysis indicated that mutations in G646W or *RUNX1* co‐mutations were independently associated with a dismal clinical outcome in the *ASXL1* ^mt^ cohort considering age, diagnosis, co‐mutations, and mutation site. To the best of our knowledge, this is the first study to provide real‐world data that clarify an adverse prognosis of *ASXL*1‐G646W mutations in patients with AML and MDS, which sheds light on a new perspective of prognostic risk factors in patients with *ASXL1* ^mt^. One of the major limitations in our study lies in withdraw bias due to the small sample size in a single center. Another limitation presents incomplete data as a result of a retrospective design and a short follow‐up. In addition, restricted to the minimal panel of gene mutations and fusion genes, only partial genetic features were shown in our cohort.

## AUTHOR CONTRIBUTIONS

**Liqing Yang:** Conceptualization (equal); data curation (equal); formal analysis (equal); investigation (equal); methodology (equal); resources (equal); software (equal); validation (equal); visualization (equal); writing – original draft (equal). **Xiaoyu Wei:** Conceptualization (equal); data curation (equal); formal analysis (equal); investigation (equal); methodology (equal); resources (equal); software (equal); validation (equal); visualization (equal); writing – original draft (equal). **Yuping Gong:** Conceptualization (lead); data curation (supporting); investigation (lead); project administration (lead); supervision (lead); validation (equal); writing – review and editing (lead).

## FUNDING INFORMATION

Not applicable.

## CONFLICT OF INTEREST STATEMENT

The authors report that there are no competing interests to declare.

## ETHICS APPROVAL STATEMENT

This study was performed in accordance with the principles of the Declaration of Helsinki. Approval was granted by the Ethics Committee of West China Hospital, Sichuan University (Sichuan, China).

## PARTICIPATE CONSENT STATEMENT

Informed consent was obtained from all participants enrolled in the study.

## Supporting information


Data S1:

## Data Availability

All data generated or analyzed are provided in the current article and supplemental materials. Further details are available upon request from the corresponding author.
