# Supplementary material for: Prognosis and risk factors for ASXL1 mutations in patients with newly diagnosed acute myeloid leukemia and myelodysplastic syndrome
Source: Cancer Med. 2023 Dec 26;13(1):e6871. doi: 10.1002/cam4.6871 (PMC10807681; doi:10.1002/cam4.6871)
Supplement: Supplementary file 1 — Data S1: [file CAM4-13-e6871-s001.docx]

**Table S1. Panel of 34 high-frequency gene mutations in the myeloid system**

| Gene | Transcript | Region | Gene | Transcript | Region |
| --- | --- | --- | --- | --- | --- |
| *ASXL1* | NM_015338 | Exon12 | *MPL* | NM_005373 | Exon10 |
| *BCOR* | NM_001123385 | Exon2-15 | *NF1* | NM_001042492 | Exon1-49 |
| *BCORL1* | NM_021946 | Exon1-12 | *NPM1* | NM_002520 | Exon11 |
| *CALR* | NM_004343 | Exon9 | *NRAS* | NM_002524 | Exon2, 3 |
| *CBL* | NM_005188 | Exon8, 9 | *PHF6* | NM_032458 | Exon2-10 |
| *CEBPA* | NM_004364 | Exon1 | *PIGA* | NM_002641 | Exon2-6 |
| *CSF3R* | NM_156039 | Exon14-17 | *PTPN11* | NM_002834 | Exon3, 8+9, 12, 13 |
| *DNMT3A* | NM_175629 | Exon2-23 | *RUNX1* | NM_001754 | Exon2-9 |
| *ETV6* | NM_001987 | Exon1-8 | *SETBP1* | NM_015559 | Exon4 |
| *ETNK1* | NM_018638 | Exon3 | *SF3B1* | NM_012433 | Exon12-15 |
| *EZH2* | NM_004456 | Exon2-20 | *SRSF2* | NM_001195427 | Exon1 |
| *FLT3* | NM_004119 | Exon20 | *STAG2* | NM_001042749 | Exon2-33 |
| *IDH1* | NM_005896 | Exon4 | *TET2* | NM_001127208 | Exon3-11 |
| *IDH2* | NM_002168 | Exon4 | *TP53* | NM_000546 | Exon2-11 |
| *JAK2* | NM_004972 | Exon12-16,20+21 | *U2AF1* | NM_006758 | Exon2, 6 |
| *KIT* | NM_000222 | Exon2, 8-11, 13, 17 | *WT1* | NM_024426 | Exon7, 9 |
| *KRAS* | NM_033360 | Exon2, 3 | *ZRSR2* | NM_005089 | Exon1-11 |

Abbreviation: *BCOR*- BCL6 Corepressor; *BCORL1*-BCL6 corepressor like 1; *CALR*-Calreticulin; *CBL*-Cbl Proto-Oncogene; *CEBPA*-CCAAT Enhancer Binding Protein Alpha; *CSF3R*-Colony Stimulating Factor 3 Receptor; *DNMT3A*-DNA Methyltransferase 3 Alpha; *ETV6*-ETS Variant Transcription Factor 6; *ETNK1*-Ethanolamine Kinase 1; *EZH2*-Enhancer of Zeste Homolog 2; *FLT3*-FMS Related Receptor Tyrosine Kinase 3; *IDH1/2*-Isocitrate Dehydrogenase 1/2; *JAK2*-Janus Kinase 2; *KIT*-KIT Proto-Oncogene, receptor tyrosine kinase; *KRAS*-Kirsten Rat Sarcoma Viral Oncogene Homolog; *MPL*-MPL Proto-Oncogene, Thrombopoietin Receptor; *NF1*-Neurofibromin 1; *NPM1*-Nucleophosmin 1; *NRAS*-Neuroblastoma RAS Viral Oncogene Homolog; *PHF6*-PHD Finger Protein 6; *PIGA*-Phosphatidylinositol Glycan Anchor Biosynthesis Class A; *PTPN11*-Protein Tyrosine Phosphatase Non-Receptor Type 11; *RUNX1*-RUNX Family Transcription Factor 1; *SETBP1*- SET Binding Protein 1; *SF3B1*-Splicing Factor 3b Subunit 1; *SRSF2*-Serine and Arginine Rich Splicing Factor 2; *STAG2*-Stromal Antigen 2; *TET2*-Ten-Eleven Translocation Methylcytosine Dioxygenase 2; *TP53*-Tumor Protein P53; *U2AF1*-U2 Small Nuclear RNA Auxiliary Factor 1; *WT1*-Wilms Tumor Gene 1; *ZRSR2*-Zinc Finger CCCH-Type, RNA Binding Motif and Serine/Arginine Rich 2.

**Table S2. Additional clinical characteristics** **in patients with *ASXL1*^mt^**

|  | ***ASXL1*^wt^ (n=185)** | ***ASXL1*^mt^ (n=34)** | ***P*-value** |
| --- | --- | --- | --- |
| **AML Cohort** | 157 (84.86) | 19 (55.88) | <0.001^§^ |
| ^a^Secondary AML | 6 (3.82) | 3 (15.79) | 0.092^§^ |
| **Morphological Subtypes (1976 FAB)** | | | |
| M1 | 28 (17.83) | 0 | 0.123^¶^ |
| M2 | 78 (49.68) | 12 (63.16) |  |
| M4 | 38 (24.20) | 5 (26.32) |  |
| M5 | 9 (5.73) | 2 (10.53) |  |
| Other subtypes | 4 (2.55) | 0 | NA |
| **Risk Stratification (2022 ELN)** | | | |
| Favorable | 21 (13.38) | 0 | NA |
| Intermediate | 86 (54.78) | 0 | NA |
| Adverse | 50 (31.85) | 19 (100.00) | <0.001^¶^ |
| **MDS Cohort** | 28 (15.14) | 15 (44.12) | <0.001^§^ |
| Transformation into AML | 7 (25.00) | 1 (4.00) | 0.289^§^ |
| **Morphological Subtypes (5th WHO)** | | | |
| IB1 | 12 (42.86) | 8 (53.33) | 0.540^§^ |
| IB2 | 16 (57.141) | 7 (46.67) |  |
| **Risk Stratification in the (2012 IPSS-R)** | | | |
| Very Low | 1 (3.57) | 0 | NA |
| Low | 0 | 0 | NA |
| Intermediate | 6 (21.43) | 3 (20.00) | 0.073^¶^ |
| High | 7 (25.00) | 9 (60.00) |  |
| Very High | 14 (50.00) | 3 (20.00) |  |
| **Treatment (1st Induced Therapy)** | | | |
| HMA-Included | 62 (37.58) | 13 (61.90) | 0.037^§^ |
| HMA alone | 8 (12.90) | 5 (38.46) | 0.166^¶^ |
| HMA+IA/DA included | 24 (38.71) | 3 (23.08) |  |
| HMA+HA included | 5 (8.06) | 2 (15.38) |  |
| HMA+venclexta included | 18 (29.03) | 3 (23.08) |  |
| Others | 11 (17.74) | 0 | NA |
| HMA-Excluded | 103 (62.42) | 8 (38.10) | 0.037^§^ |
| IA/DA included | 89 (86.41) | 7 (87.50) | 1.000^§^ |
| HA included | 10 (9.71) | 1 (12.50) | 1.000^§^ |
| Venclexta alone | 2 (1.94) | 0 | NA |
| Others | 2 (1.94) | 0 | NA |

Statistical analysis: ^§^(approximate) chi-square test, ^¶^Fisher's exact test.

Abberations: IA-Combined treatment of darubicin and cytarabine; DA-Combined treatment of daunorubicin and cytarabine; HA-Combined treatment of homoharringtonine and cytarabine.

^a^sAML could be arising post other myeloid neoplams besides MDS.

**
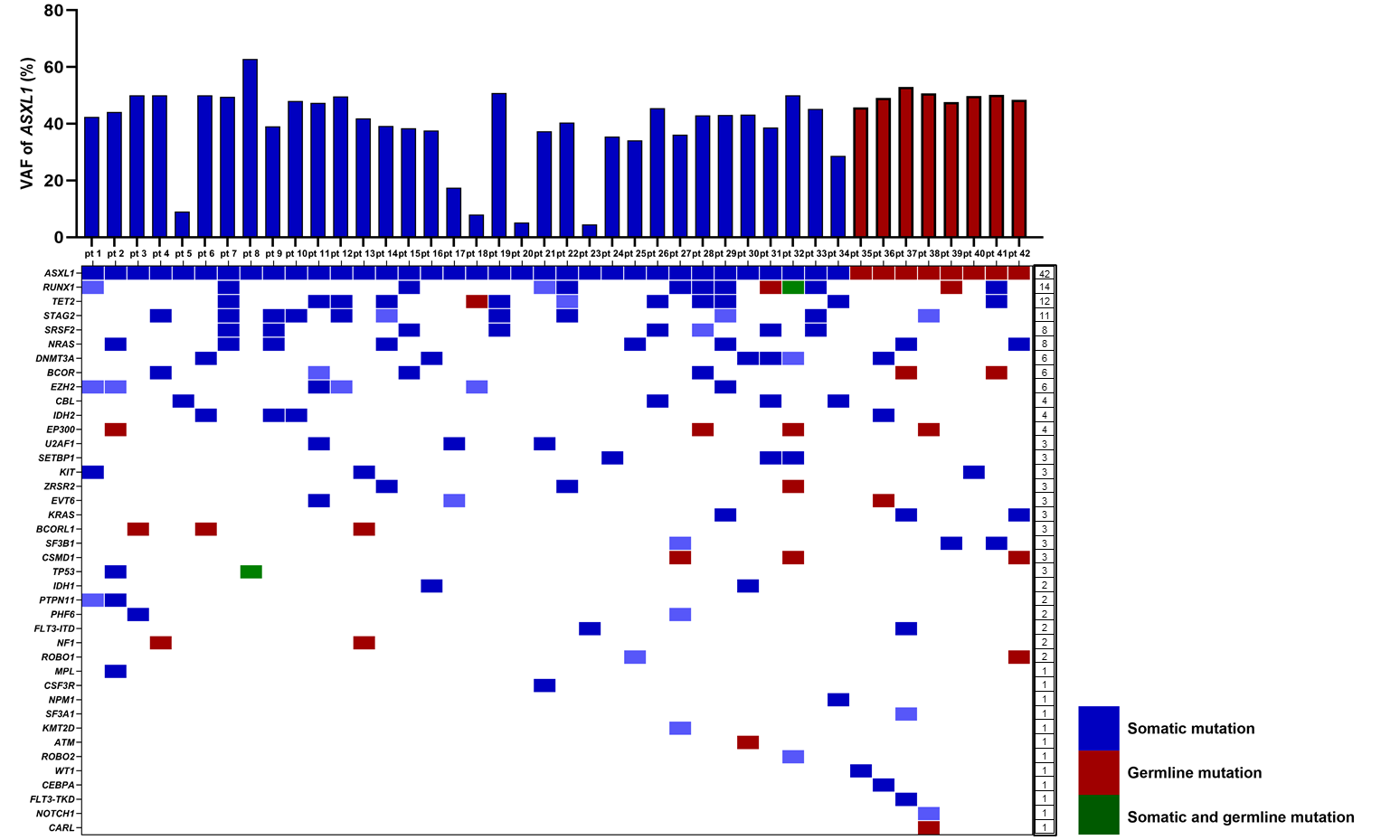
**

**Figure S1. Comutations in patients with *ASXL1*^mt^ (n=34)**

a
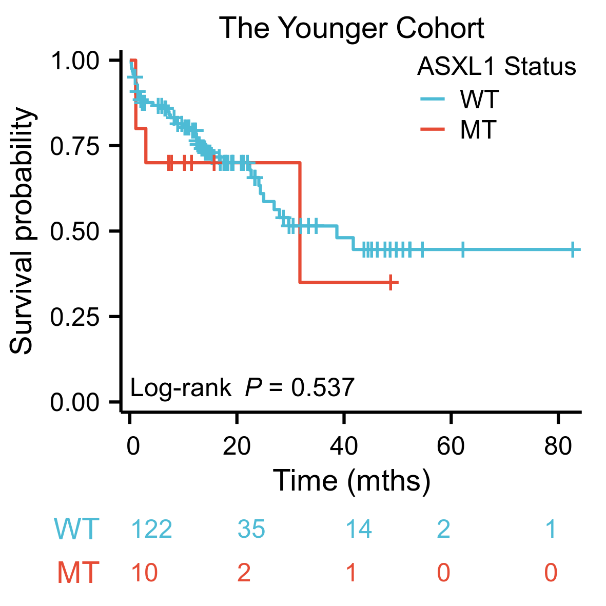
 b
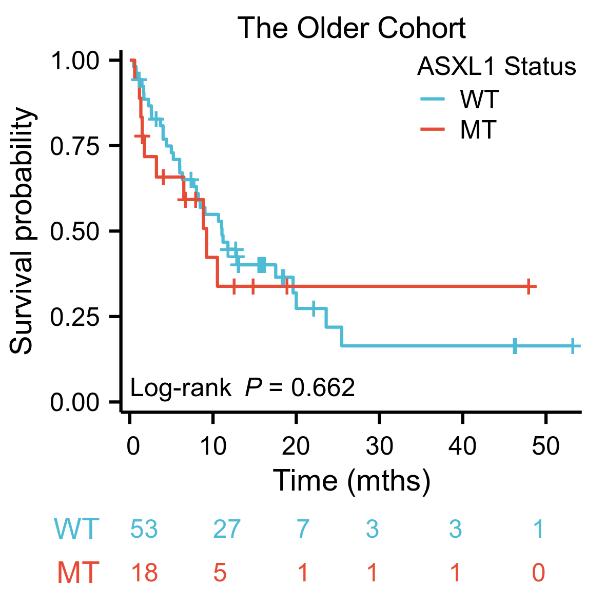
c
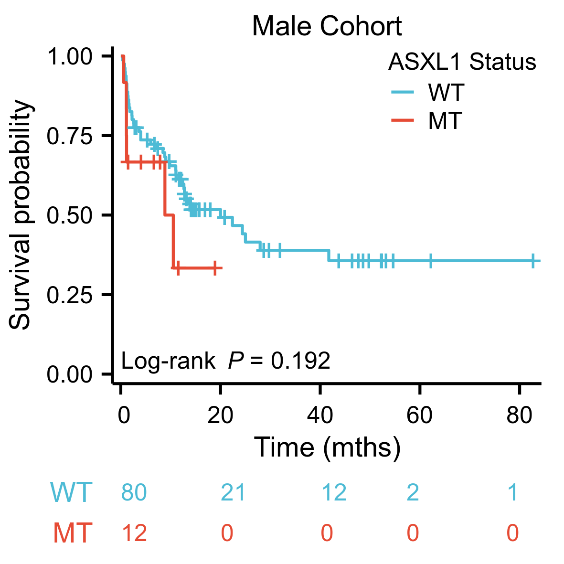
d
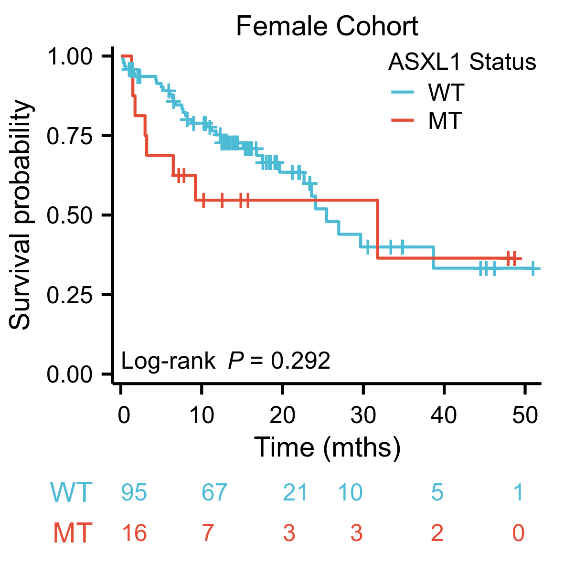
 e
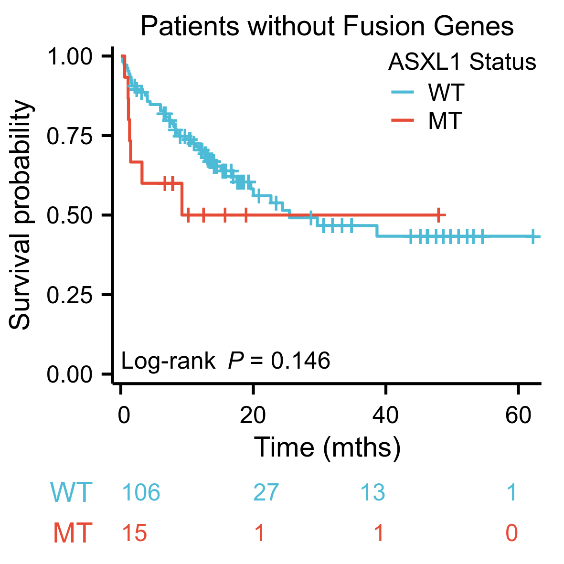
f
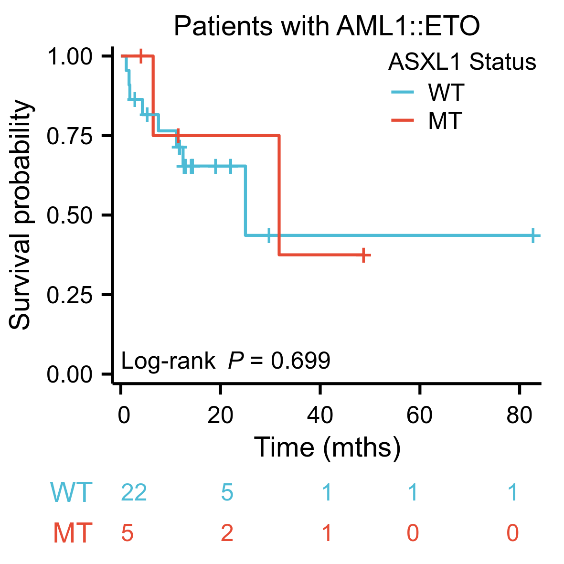
g
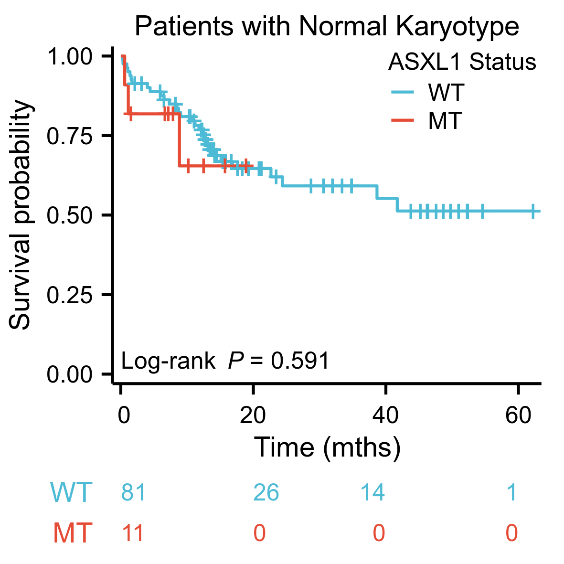
h
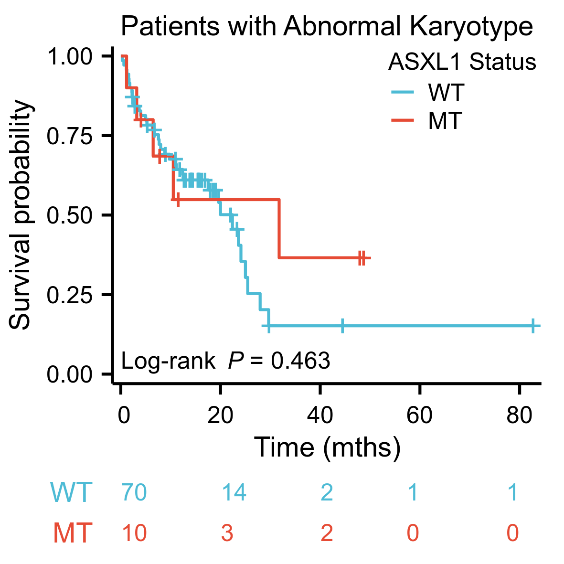
 i
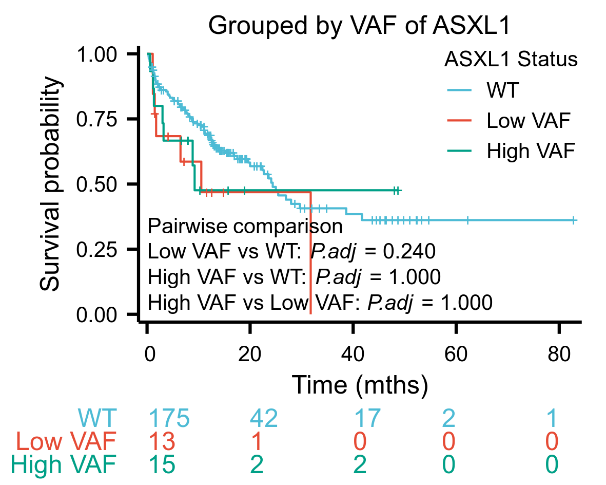
j
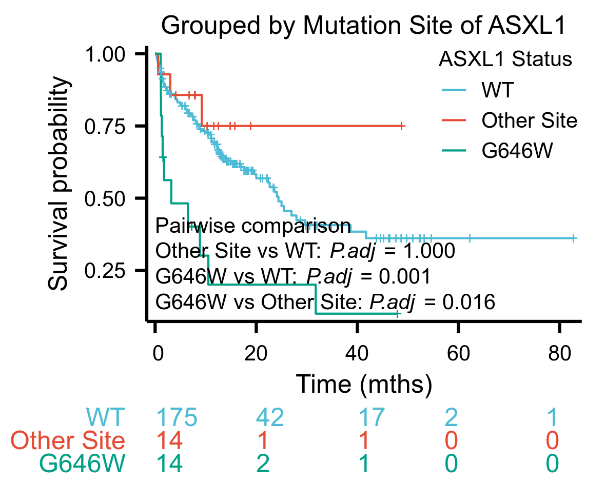
k
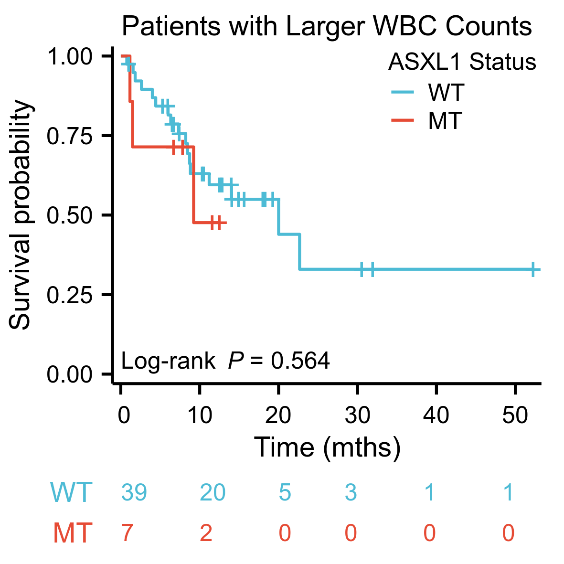
 l
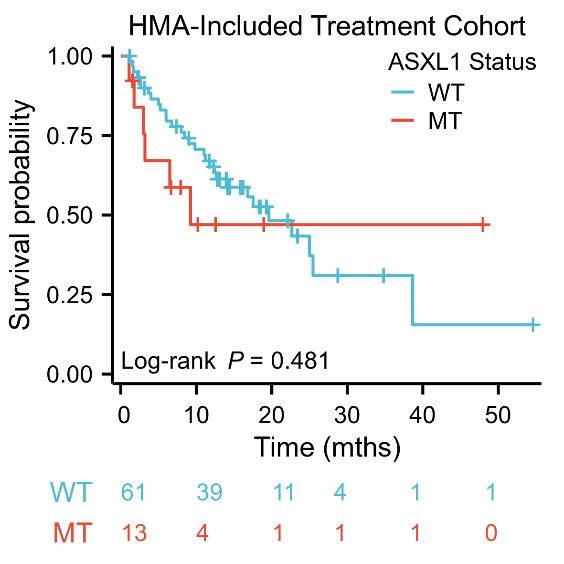


**Figure S2. Survival analysis based on clinical and genetic characteristics**

The optimal cut-off values of age (58-year-old) in Figure S2-a&b and the WBC count (35×10^7^/L) in Figure S2-k were determined by the ROC curve.

The cut-off value of VAF in Figure S2-i was based on the median.

a
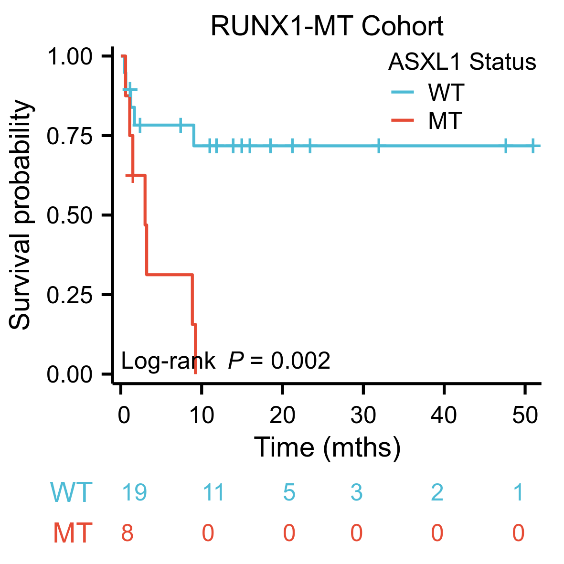
 b
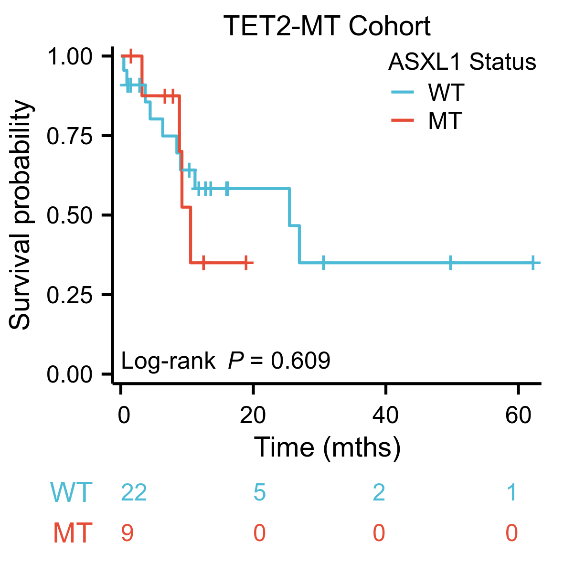
c
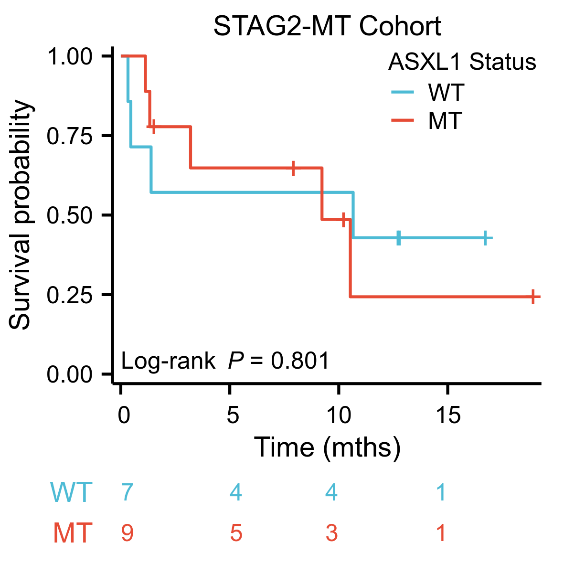
 d
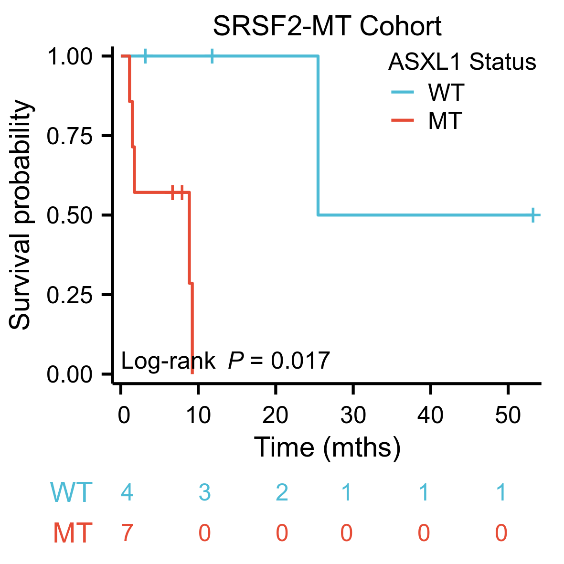
 e
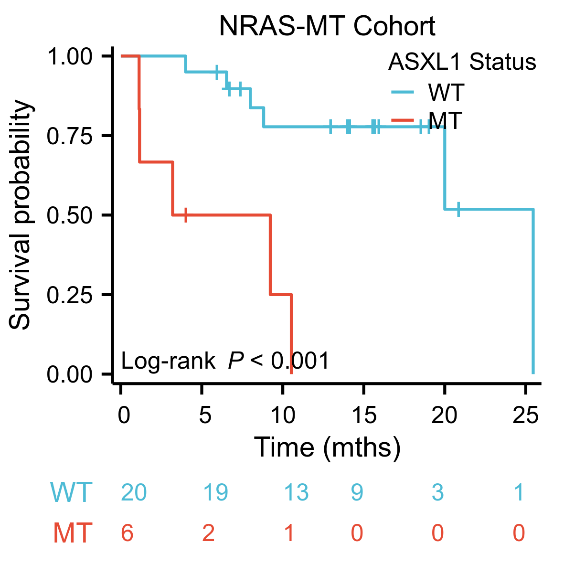
 f
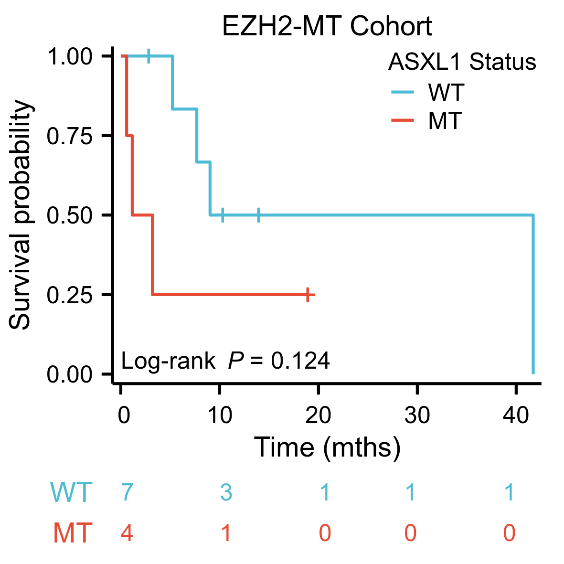
g
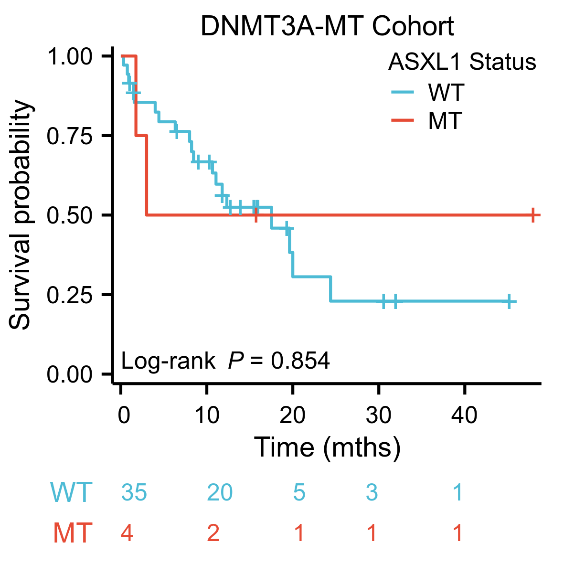


**Figure S3. ​Survival analysis based on comutations of high frequencies.**

**
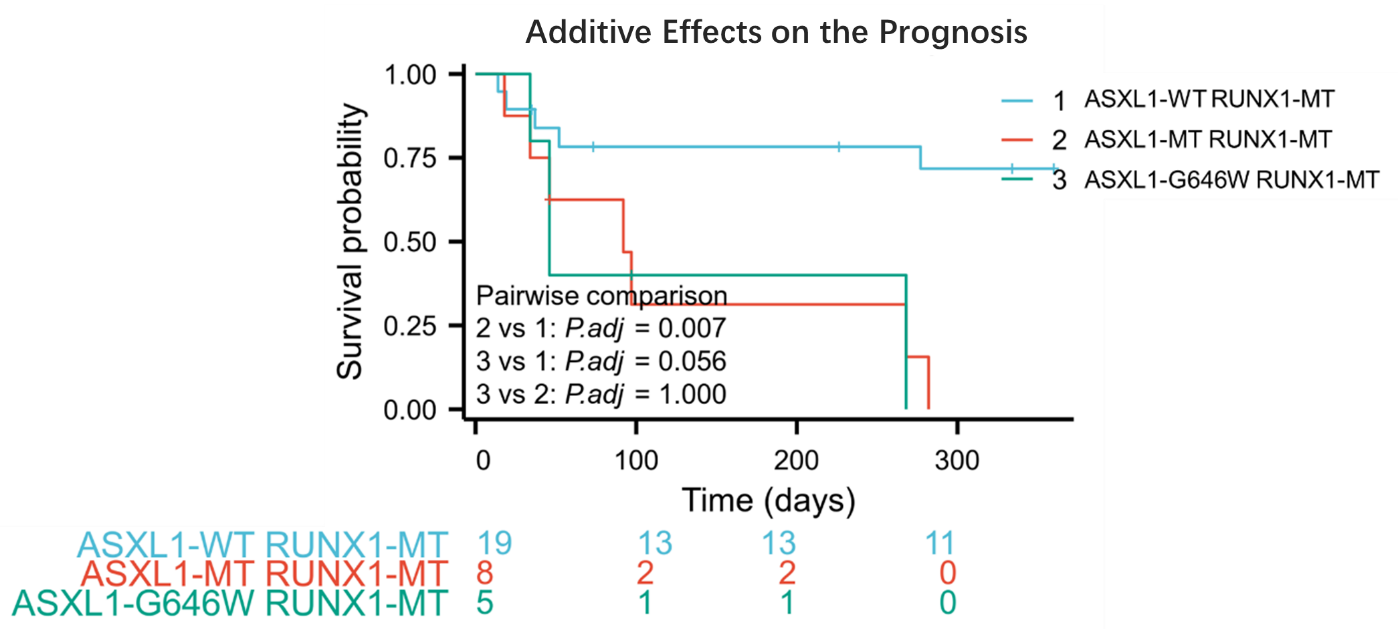
**

**Figure S4. Survival analysis of the** **additive effects of *ASXL1*^G646W^ in patients with *RUNX1*^mt^**

**Table S3. Treatment and clinical outcomes of patients with monocytic AML**

|  | ***ASXL1*^wt^ (n=46)** | ***ASXL1*^mt^**  **(n=7)** | ***P*-value** |
| --- | --- | --- | --- |
| **Age (mean ± SD)** | 47.67±2.63 | 56.00±7.29 | 0.259 |
| **Male (n, %)** | 17 (36.96) | 3 (42.86) | 1.000^§^ |
| **AML-M4 (n, %)** | 37 (80.43) | 5 (71.43) | 0.962^§^ |
| **AML-M5 (n, %)** | 9 (18.57) | 2 (28.57) |  |
| **Treatment (n, %)** | 42 (91.30) | 5 (71.43) | 0.365^§^ |
| HMA-included | 9 (21.43) | 2 (40.00) | 0.713^§^ |
| HMA+IA/DA | 3 (33.33) | 0 | NA |
| HMA+venclexta included | 6 (66.67) | 2 (100.00) | NA |
| HMA-excluded | 33 (78.57) | 3 (60.00) | 0.713^§^ |
| IA/DA included | 31 (93.94) | 3 (100.00) | NA |
| HA included | 2 (6.06) | 0 | NA |
| **Response (n, %)** |  |  |  |
| Complete Remission | 33 (78.57) | 3 (60.00) | 0.713^§^ |
| Relapse | 13 (39.39) | 1 (33.33) | 1.000^§^ |
| Death | 16 (34.78) | 2 (28.57) | 1.000^§^ |

Statistical analysis: ^§^(approximate) chi-square test

**
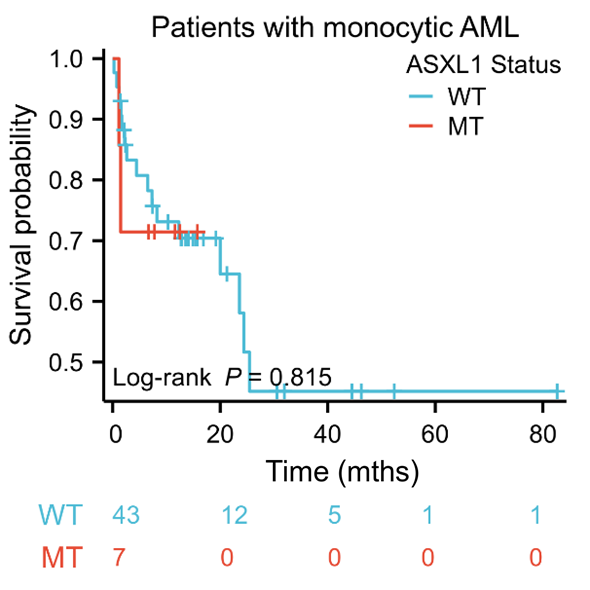
**

**Figure S5. Survival analysis in patients with monocytic AML**
